# Ancestral State Reconstruction of the Apoptosis Machinery in the Common Ancestor of Eukaryotes

**DOI:** 10.1534/g3.118.200295

**Published:** 2018-04-27

**Authors:** Joanna Klim, Arkadiusz Gładki, Roza Kucharczyk, Urszula Zielenkiewicz, Szymon Kaczanowski

**Affiliations:** * Department of Microbial Biochemistry; † Department of Bioinformatics; ‡ Department of Genetics, Institute of Biochemistry and Biophysics, Polish Academy of Sciences, 02-106, Pawińskiego 5a Warsaw, Poland

**Keywords:** evolution, phylogenetics, ancestral state reconstruction, apoptosis, mitochondrial domestication

## Abstract

Apoptotic cell death is a type of eukaryotic cell death. In animals, it regulates development, is involved in cancer suppression, and causes cell death during pathological aging of neuronal cells in neurodegenerative diseases such as Alzheimer’s. Mitochondrial apoptotic-like cell death, a form of primordial apoptosis, also occurs in unicellular organisms. Here, we ask the question why the apoptosis machinery has been acquired and maintained in unicellular organisms and attempt to answer it by performing ancestral state reconstruction. We found indications of an ancient evolutionary arms race between protomitochondria and host cells, leading to the establishment of the currently existing apoptotic pathways. According to this reconstruction, the ancestral protomitochondrial apoptosis machinery contained both caspases and metacaspases, four types of apoptosis induction factors (AIFs), both fungal and animal OMI/HTR proteases, and various apoptotic DNases. This leads to the prediction that in extant unicellular eukaryotes, the apoptotic factors are involved in mitochondrial respiration and their activity is needed exclusively in aerobic conditions. We test this prediction experimentally using yeast and find that a loss of the main apoptotic factors is beneficial under anaerobic conditions yet deleterious under aerobic conditions in the absence of lethal stimuli. We also point out potential medical implications of these findings.

Eukaryotic apoptotic cell death is a fundamental mechanism regulating multicellular development, and its origin is a fundamental question in biology.

Apoptosis was first described in animals. Its morphological and biochemical hallmarks allow one to easily distinguish it from other types of cell death (Kerr *et al.*, 1972). Apoptosis is usually initiated by mitochondrial membrane permeability transition reflected by the breakdown of the inner mitochondrial transmembrane potential. The next stage is characterized by chromatin condensation and nuclear fragmentation. Eventually the cell breaks down into membrane-surrounded fragments that are ingested by macrophages, which prevents induction of inflammation. Classical studies of Horvitz and Sulston showed that animal apoptosis is a form of programmed cell death (Sulston and Horvitz 1977; Hedgecock *et al.*, 1983). Apoptosis plays a key role in development, and homeostasis, and in preventing carcinogenesis (Kaczanowski *et al.*, 2016). Interestingly, regulated necrosis of animals cells is usually also initiated by a mitochondrial membrane permeability transition. However, during regulated necrosis the cell does not break into fragments but undergoes cellular leakage instead (Vanden Berghe *et al.*, 2014).

For a long time apoptosis was believed to occur only in animals. However, cell death similar to apoptosis has been described both in unicellular and multicellular non-animal eukaryotes. In older papers, this phenomenon was described as apoptosis-like cell death. Currently, it is referred to as mitochondrial apoptosis, or simply apoptosis (Carmona-Gutierrez *et al.*, 2018). Regulated cell death similar to animal apoptosis has been described in plants, slime molds and even in unicellular ciliates like *Tetrahymena* and even in yeast (a focus of this paper) (Kaczanowski 2016). Yeasts were used as a model organism in studies of primordial form of apoptosis (see as a review(Carmona-Gutierrez *et al.*, 2010).

There are also other types of programmed cell death not initiated by mitochondria, including programmed cell death of bacteria and amitochondrial protozoans such as *Trichomonas vaginalis* (Chose *et al.*, 2003; Chose *et al.*, 2002; Kaczanowski 2016).

It remains unclear why the apoptosis machinery has been acquired and maintained in unicellular organisms (Proto *et al.*, 2013; Taylor-Brown and Hurd 2013; Durand *et al.*, 2016; Carmona-Gutierrez *et al.*, 2012; Madeo *et al.*, 2004; Rockenfeller and Madeo 2008). Some hypothesize that apoptosis is an altruistic suicide maintained by kin selection (Carmona-Gutierrez *et al.*, 2010; Duszenko *et al.*, 2006; Kaczanowski 2016; Taylor-Brown and Hurd 2013) whereas others consider it an antagonistic pleiotropy. Garrido and Kroemer (Garrido and Kroemer 2004) pointed out that apoptotic proteins also have non-apoptotic vital functions. In yeasts mitochondrial apoptotic factors are required for proper function of this organelle. NDI1 encodes the protein that oxidizes NADH and passes electrons on to ubiquinone (Marres *et al.*, 1991), NUC1 is involved in recombination of mitochondrial DNA (Zassenhaus and Denniger 1994). Main cytoplasmic apoptotic factors MCA1 and OMI/HTRA are involved in proteolysis quality control (Clausen *et al.*, 2011; Lee *et al.*, 2010).

The apoptosis mechanisms found in different clades are similar to one another; they all involve the mitochondrion as the central player (Kroemer and Reed 2000; Büttner *et al.*, 2007; Arambage *et al.*, 2009; Kaczanowski 2016; Reape and McCabe 2010). The release of mitochondrial apoptotic factors induces self-destruction of nuclear DNA by DNases (*e.g.*, ENDOG, ZEN1, and NUC1) and cell death. Cell death can also be induced by apoptotic proteases, which include metacaspases, caspases, and OMI protease (OMI)/high temperature requirement A (HTRA) (Kaczanowski 2016).

Although the overall mechanisms of apoptosis are nearly universal, the apoptotic proteins of different taxonomic groups are often only remotely evolutionarily related, and some are not related at all. For example, the plant apoptotic DNase ZEN1 is not related to any animal apoptotic DNase, while the animal apoptotic caspases are remotely related to an analogous proteases of plants and fungi called metacaspases (Uren *et al.*, 2000; Aravind *et al.*, 2001). These observations prompt the following question: What is the evolutionary origin of apoptosis mechanisms – convergent evolution, divergent evolution, or a combination of the two? Earlier phylogenetic analyses of animal apoptosis supported the endosymbiotic theory of the origin of apoptosis, which is based on the endosymbiotic hypothesis of mitochondrial origin first postulated by Margulis (Margulis *et al.*, 2006). The endosymbiotic theory of a mitochondrial origin for apoptosis was first proposed by Kroemer (Kroemer 1997), who suggested that extant apoptotic factors are modified bacterial toxins used by the protomitochondrion before and/or during the establishment of symbiosis with protoeukaryotes. In the case of animals the endosymbiotic theory of a mitochondrial origin of apoptosis was tested in phylogenetic studies performed by Aravind, Dixit, and Koonin (Aravind *et al.*, 2001; Koonin and Aravind 2002), who concluded that pro-apoptotic factors often have eubacterial origins.

In this paper we ask if this theory is more general and could also be applied to non-animal eukaryotes. To answer this question we focus on the mentioned basic mechanisms of apoptosis shared by different taxonomic groups and restrict our analysis to the ancient cytoplasmic and mitochondrial factors involved directly in apoptosis activation. Therefore, we exclude from the analysis proteins involved in permeability transition and mitochondrial stability. Such proteins do not induce apoptosis directly, but rather cause the release of apoptotic factors from mitochondria.

We take advantage of the recent advances in systematics that have revealed that the six to eight major eukaryotic branches appeared very early in evolutionary history (Adl *et al.*, 2012; del Campo *et al.*, 2014). Our results are based on genomic data from the following major eukaryotic groups: Opisthokonta (fungi and animals); Amoebozoa (*Dictyostelium*); SAR–Stramenopiles, Alveolates, and Rhizaria –(ciliates, apicomplexan parasites and *Reticulomyxa*); Excavata (kinetoplastids, *Trichomonas*, *Naegleria*); and Archaeplastida (plants and green algae such as *Volvox*). Using a parsimony assumption, we reason that an apoptosis factor was part of the ancestral machinery if it has homologs in organisms belonging to several of these ancient taxonomic groups, or in non-eukaryotic organisms. A phylogenetic analysis of the relationship with eubacterial factors was used for the identification of putative ancient duplications predating the domestication of mitochondria. Using this approach, we identified the ancestral apoptotic factors.

Our analysis also confirmed that apoptosis evolved during the domestication of mitochondria. Indeed, we found indications of an ancient evolutionary arms race between protomitochondria and host cells, leading to the establishment of the extant apoptotic pathways. That observation led to an experimentally testable hypothesis that the ancient apoptotic factors originated from protomitochondrial proteins with other functions. We assumed that mitochondrial domestication was an adaptation to aerobic conditions and was beneficial exclusively in such conditions. We also posited that the apoptosis machinery was required for proper mitochondrial function and therefore was involved in adaptation to aerobic conditions. This mechanism has therefore been maintained since mitochondrial domestication until present. We tested this hypothesis experimentally using the yeast *Saccharomyces cerevisiae* and found that expression of apoptotic inducers is beneficial exclusively in aerobic conditions.

## Materials and Methods

Nine Pfam domains were studied (see details in Table 1) using an our own pipeline. Using this pipeline, we identified members of the families from different genomic databases, which were not present in the Uniprot database. Using BLAST software, we removed redundant sequences, which were present in both the Uniprot and genomic databases. It was also used to prepare alignments that are used for phylogenetics. Using this approach, we obtained confident alignments that contain thousands of sequences. Detailed description is presented below.

**Table 1:** Pfam domains selected for study

Domain ID	Name of primary orthologous group	Number of protein sequences (in the final tree and alignment)
PF07992	AIF	48731 (smaller set 12420)
PF02037	API5	177
PF01027	BI	5378
PF00653	BIR	1748
PF00656	Caspase/Metacaspase	3521
PF01223	ENDOG	2027
PF03265	NUC1	396
PF13180	OMI	11284

For each domain, full alignments were fetched from the Pfam database. The Sreformat tool was used to convert the Stockholm format to the FASTA alignment format. As a part of the quality control procedure, for each alignment, sequences from species absent in the UniProt taxonomy were filtered out. Then, HMM profiles (Eddy 2011) and BLAST (Altschul *et al.*, 1990) databases were created from each preprocessed alignment.

The HMM profiles obtained in the previous step were used to find all the proteins (with the use of the hmmsearch tool from HMMER3 package) containing a given Pfam domain in the predicted proteomes of the selected species: *Arabidopsis thaliana*, *Caenorhabditis elegans*, *Chlamydomonas reinchardtii*, *Dictyostelium discoideum*, *Drosophila melanogaster*, *Homo sapiens*, *Leishmania major*, *Naegleria gruberi*, *Plasmodium falciparum*, *Reticulomyxa filose*, *Saccharomyces cerevisiae*, *Schizosaccharomyces pombe*, *Toxoplasma gondii*, *Trichomonas vaginalis*, *Trypanosoma brucei*, *Trypanosoma cruzi*, *Volvox carteri*, *Paramecium tetraurelia* and *Tetrahymena thermophila*, retrieved from the NCBI except for *N. gruberi* and *P. falciparum*, where the data were from JGI and PlasmoDB, respectively.

The sequences found comprised the input for subsequent BLAST searches, whereas the original but preprocessed, as described above sequences containing a given Pfam family comprised the BLAST database. With such an approach, novel sequences containing the given Pfam domain were found defined as query sequences with identity to any sequence from the BLAST database below 90% and with at least 50% coverage.

The final alignment representing each domain was created by merging the novel sequences with the original (preprocessed) ones from the Pfam alignments of a given domain. As a part of quality control, all positions with at least one insertion were removed from the alignments. Moreover, positions assigned to insertion states (represented by small letters), as well as those assigned to the N and C states, were also removed.

Finally, the modified alignments were used to create phylogenetic trees with FastTree (Price *et al.*, 2009). Both final alignments and final trees are available in Additional file 1. Alignments of representative sequences were made using MSAProbs software (Liu and Schmidt 2014), and phylogenetic trees of representative sequences were calculated using MEGA software (Tamura *et al.*, 2013).

### Description of BLAST Searches

BLAST searches were performed with the NCBI BLAST server. Two different search strategies were applied. In the first strategy, one proteome of the following 14 eukaryotic organisms was searched: *A. thaliana*, *V. carteri*, *D. discoideum*, *S. cerevisiae*, *H. sapiens*, *D. melanogaster*, *C. elegans*, *T. gondii*, *P. tetraurelia*, *T. thermophila*, *R. filose*, *T. brucei*, *T. cruzi*, and *T. vaginalis*. We called this approach the eukaryotic strategy. The second, extended strategy, along with the proteomes from the first search strategy, included all archaeal and eubacterial proteomes. The applied strategies are stored in the directory **search strategies (**Additional file 2).

### Nomenclature

Since the present study considers proteins from diverse clades the short-form abbreviations of protein nomenclature following the Pfam database style are used throughout the text, with the exception of the yeast experiments section. Standard genetic nomenclature is used to designate *S. cerevisiae* wild-type alleles (*e.g.*, *HIS3*), recessive mutant alleles (*e.g.*, *ade2–1*) and deletions (*e.g.*, *ndi1Δ*::*KANMX4)*,which means that the *NDI1*ORF has been replaced by *KANMX4*, a gene conferring G418 resistance.

### Yeast Growth Experiments

The MR6 reference strain (*MAT****a****ade2-1 his3-1,15 trp1-1 leu2-3,112 ura3-1 CAN1 arg8*::*HIS3*) and its derivatives *ndi1Δ*::*KANMX4*, *nuc1Δ*::*KANMX4*, *mca1Δ*::*KANMX4*, and *nma111Δ*::*KANMX4*, and ρ^0^ (devoid of mitochondrial DNA) MR6/b-3 (Godard *et al.* 2011) were used. Standard YPD medium (2% glucose, 1% Bacto yeast extract, 2% Bacto peptone) enriched with 40 mg/L of adenine was used. Under anaerobic conditions, the medium was supplemented with 0.5% ergosterol and Tween80 solution (1 g of ergosterol was dissolved in a mixture of ethanol and Tween80 (volume ratio 5:1)). Growth was monitored via optical density (OD_600_) measurements and viability of cells was tested by plate assays. Competition tests were performed as follows: yeast were grown overnight at 28° with shaking, adjusted to identical optical density, and then mixed at a 1:1 ratio in fresh N_2_-saturated medium or O_2_-containing medium, respectively. Co-cultures were placed in an anaerobic chamber or in aerobic conditions, accordingly and incubated at 28°. A new round of subculturings were begun by transferring proper co-culture volumes into fresh medium to obtain OD_600_ = 0.1 and then growth was continued under the described above conditions. The procedures were repeated every 24 hr, during which an average of two or three cell divisions occurred in anaerobic cultures and five to six in aerobic ones. Aliquots of appropriate dilutions of each passage were plated in triplicate on YPD plates, and 100 colonies were tested for geneticin resistance (deletion mutants) or incapability of growth on non-fermentable carbon source (ρ^0^ strain) by replicating them onto selective plates (YPD supplemented with 200 µg/ml of geneticin G418) or YPG plates (2% glycerol, 1% Bacto yeast extract, 2% Bacto peptone).

For the yeast growth curves, single colonies were grown overnight in biological duplicates in liquid YPD medium at 28° with agitation. Aliquots of the overnight cultures were inoculated to acquire OD_600_ = 0.1, into fresh medium prepared accordingly to O_2_-free or O_2_-containing experiment conditions. Growth curves were obtained at 28° in anaerobic or aerobic conditions, and optical density (OD_600_) was measured (NanoPhotometer NP80) at appropriate time intervals for about 150 h. Aliquots of appropriate dilutions of each measurement were plated in triplicate on YPD plates, and colonies were counted to estimate the number of live cells (CFU).

### Data availability

The authors state that all data necessary for confirming the conclusions presented in the article are represented fully within the article. Supplemental material available at Figshare https://doi.org/10.6084/m9.figshare.5548519.

## Results

### Core of Yeast Apoptosis Machinery Is Reduced to Five Proteins

We explored the Saccharomyces Genome Database (SGD) to identify the core apoptosis machinery. There are 31 known proteins a lack of which decreases apoptotic activity and 13 proteins whose overexpression induces apoptosis in diverse experimental conditions. Four proteins are shared by these two sets: apoptotic protease metacaspase MCA1, apoptotic nuclease NUC1, and apoptotic induction factors NDI1 and AIF1. Three of these proteins, NDI1, AIF1 and NUC1, have mitochondrial localization, and MCA1 is cytosolic. We included additionally to this core HTRA/Omi protease, because according to literature overexpression of HTRA-encoding gene induces apoptosis and its deletion suppresses apoptosis (Fahrenkrog *et al.*, 2004). All these proteins are involved in apoptosis induced by H_2_O_2_ and occurring in chronological aging (Büttner *et al.*, 2007; Herker *et al.*, 2004; Madeo *et al.*, 2004; Madeo *et al.*, 2002; Fahrenkrog *et al.*, 2004; Walter *et al.*, 2006; Li *et al.*, 2006; Cui *et al.*, 2012). Interestingly, it was shown that mitochondrial metabolism has an impact on apoptotic activity of NUC1 factor. Deletion of NUC1 suppress apoptotic cell death when yeasts are cultured on non-fermentable sources of carbon sources and in result mitochondrial respiration is stimulated. In contrast deletion of NUC1 strongly stimulate necrotic cell death when oxidative phosphorylation is repressed. (Büttner *et al.*, 2007). Investigated apoptotic factors are involved in different apoptotic pathways. It has been shown that apoptosis induced by NUC1 and NDI1-encoded factors are metacaspase independent (Cui *et al.*, 2012; Büttner *et al.*, 2007).

This analysis confirmed our recently published model of an evolutionarily conserved ‘core apoptosis machinery’ reduced to three groups of apoptotic inducers: proteases, nucleases and apoptotic factors involved in respiration (Kaczanowski 2016). Additionally, it shows that the apoptosis mechanisms used by extant unicellular organisms are much simpler than the animal ones and are reduced to this conserved core. Further we focus on the evolution of proteins from these three groups.

### Ancient Apoptotic DNases

**Figure 1:**
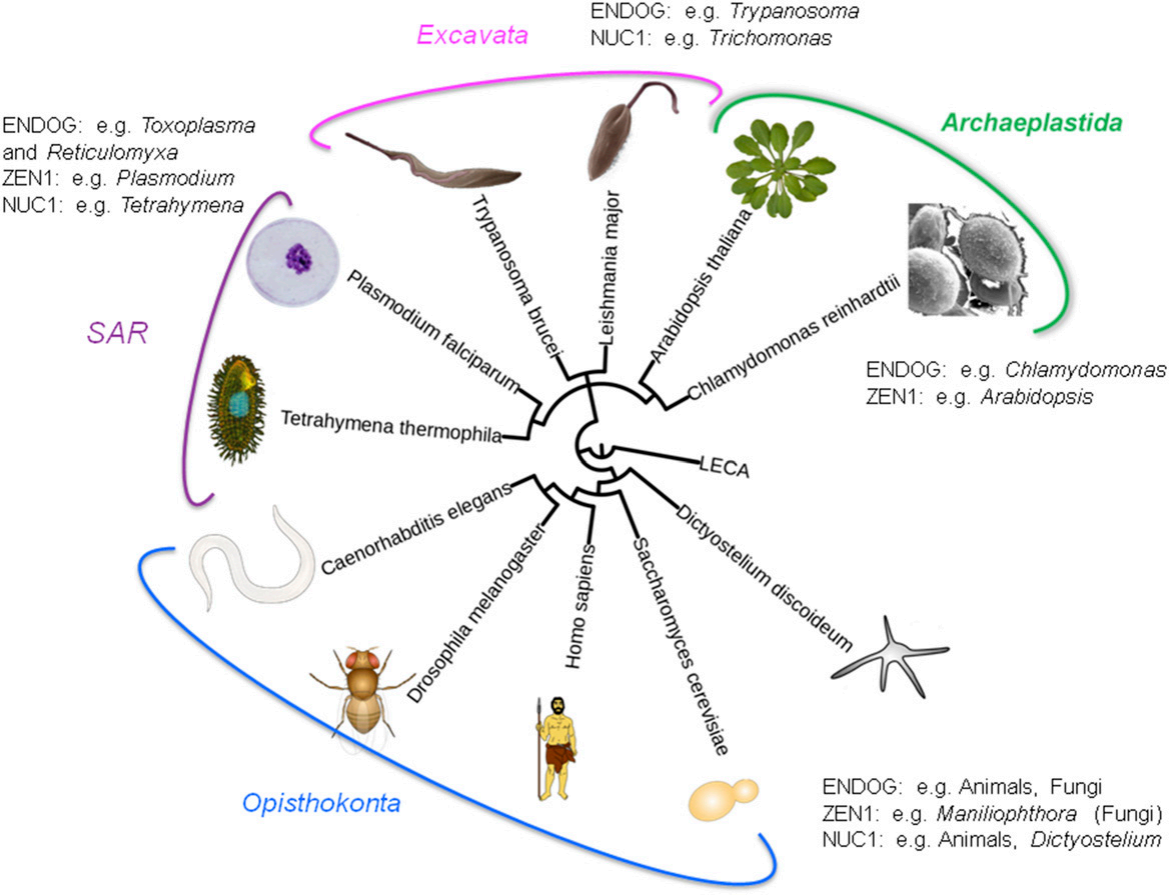
Evolutionary tree of eukaryotes and of apoptotic DNases. The phylogenetic tree is based on a recently published analysis of the evolution of 37 proteins and shows organisms with described apoptosis. The authors obtained bootstrap values of 100% for the presented branches downloaded from Wikipedia (https://commons.wikimedia.org/wiki/File:Budding_yeast_(Saccharomyces_cerevisiae).png https://commons.wikimedia.org/wiki/Category:Caenorhabditis_elegans#/media/File:201108_nematode.png https://en.wikipedia.org/wiki/Trypanosoma_brucei#/media/File:TrypanosomaBrucei_ProcyclicTrypomastigote_SEM.jpg https://commons.wikimedia.org/wiki/File:Homosapiens.svg https://commons.wikimedia.org/wiki/File:Arabidopsis_thaliana_rosette_transparent_background.png https://commons.wikimedia.org/wiki/Category:Leishmania_mexicana#/media/File:LeishmaniaMexicana_Promastigote_SEM.jpg https://commons.wikimedia.org/wiki/File:Plasmodium_malariae_01.png https://creativecommons.org/))license.

There appear to be three ancient DNases related to apoptosis: ENDOG, ZEN1 and NUC1 (Kaczanowski 2016; Kaczanowski *et al.*, 2011). The apoptotic function of ENDOG, amitochondrial DNase, has been demonstrated in organisms belonging to two ancient eukaryotic phylogenetic branches, the Opisthokonta comprising yeast (Büttner *et al.*, 2007) and animals (Li *et al.*, 2001), and the Excavata (trypanosomes) (Gannavaram *et al.*, 2008). The fact that these three proteins evolved very early, as the data for ENDOG suggests, was confirmed by analysis based on the eukaryotic evolutionary tree shown in Figure 1.

ENDOG proteins contain the Protein Families Database (Pfam) Endonuclease_NS (ID PF01223) domain (DNA/RNA non-specific domain), which is mainly present in eukaryotic and eubacterial proteins (some archaeal and viral proteins contain this domain as well). Maximum likelihood phylogenetic trees revealed that ENDOG proteins belong to a monophyletic branch that includes eubacterial (but not archaeal) proteins, suggesting that the ENDOG protein has a mitochondrial/eubacterial origin. This hypothesis is supported by the fact that ENDOG is a mitochondrial protein. This branch also contains ENDOG homologs encoded in the genomes of organisms belonging to major eukaryotic taxonomic groups, besides two mentioned earlier, namely SAR (*Toxoplasma* and *Reticulomyxa*) and Archaeplastida (*Volvox* and *Chlamydomonas*). Thus we conclude that ENDOG was part of the protomitochondrial apoptosis system.

Similar analyses were performed for the ZEN1 and NUC1 proteins using examples indicated in Figure 1. The apoptotic function of ZEN1 DNase has been described in plants (Ito and Fukuda 2002). ZEN1 contains the S1-P1 nuclease domain (ID PF00265), which is present almost exclusively in eukaryotes and eubacteria (there are also examples in eukaryotic viruses). An inspection of the phylogenetic tree indicated that the branch containing plant ZEN1 proteins contains eubacterial homologs and proteins from Opisthokonta (fungi, *e.g.*, *Moniliophthora*) and SAR (Apicomplexa, *e.g.*, *Plasmodium*).

The NUC1 protein is one of the animal apoptotic DNases (Lyon *et al.*, 2000). It contains the Pfam domain DNase II ID PF03265 which is also present in the proteins of eubacteria and viruses but not in those of Archaea. As with ENDOG and ZEN1, homologs of the NUC1 occur in several major eukaryotic groups, including SAR (ciliates and *Reticulomyxa*), Excavata (*Trichomonas*) and Amoebozoa (*Dictyostelium*). We conclude that the ancestral protomitochondrion probably had all these three apoptotic DNases, some of which were subsequently lost in various lineages.

In conclusion, the results presented above suggest that the protomitochondrion encoded all three ancient DNases: ENDOG, ZEN1 and NUC1.

### Ancient Apoptotic Proteases – Metacaspase and Caspase

**Figure 2:**
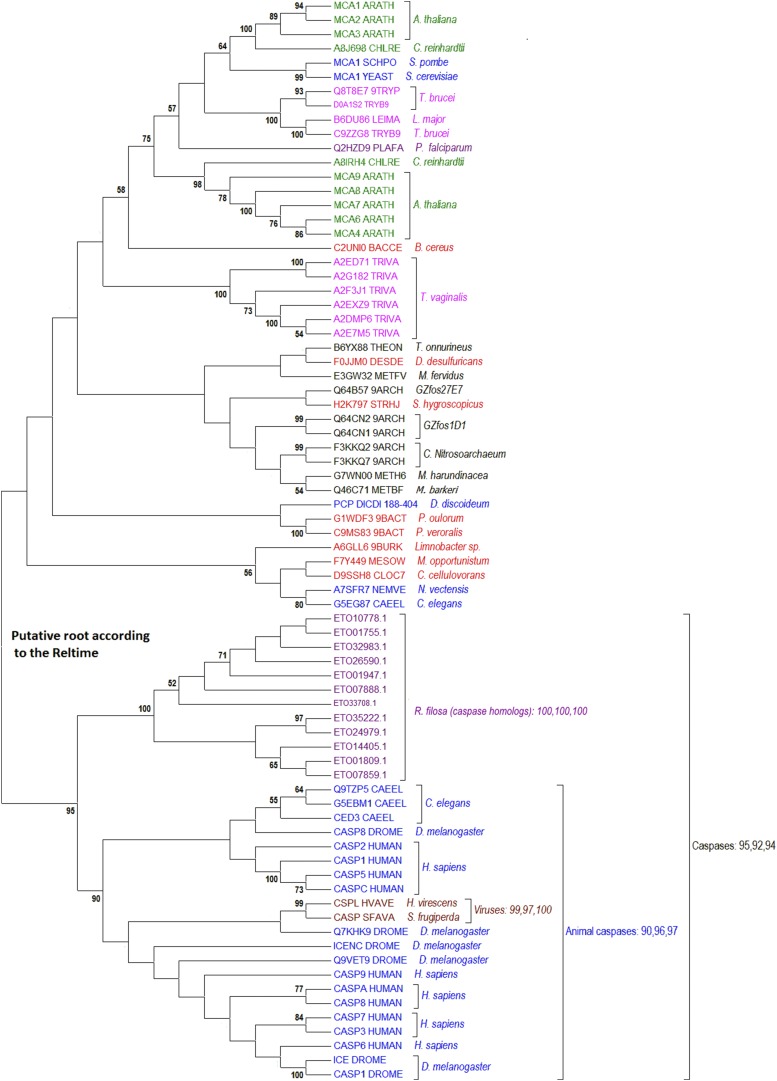
Phylogenetic tree of metacaspases/caspases. Three comma-separated bootstrap values (100 replicates) for the most significant branches were calculated with maximum likelihood estimation (MLE), neighbor-joining (NJ), and minimal evolution (ME). Red indicates bacterial proteins, black-Archaea, dark blue-Opisthokonta/Amoebozoa, green-Archaeplastida, purple-SAR, pink-Excavata, and brown-viral proteins.

In animals, apoptosis is induced by proteases called caspases (cysteine and aspartic proteases). In fungi and plants, a similar function is played by arginine and lysine-specific proteases called metacaspases. Both proteases inactivate Tudor nuclease to induce apoptosis (Sundström *et al.*, 2009). Because plants and fungi are remotely related (plants belong to Archaeplastida and fungi to Opisthokonta), we can assume that metacaspase was a part of the ancestral apoptosis machinery. Our sequence analysis suggests that caspases and metacaspases diverged at or even before the first eukaryotic cells emerged. This hypothesis is supported by three pieces of evidence. The first comes from a phylogenetic analysis of all 3521 sequences containing the peptidase C14 domain (Pfam ID PF00656), which is shared by caspases and metacaspases (see Additional file 1). The maximum likelihood tree (FastTree) has a branch containing caspases and proteases of *Reticulomyxa*, a unicellular organism and member of the SAR clade. The second piece of evidence is provided by a phylogenetic analysis based on alignment of a small number of representative sequences (Figure 2). This analysis was performed because it has been shown that errors in protein alignment may lead to errors in phylogenetic tree. The proteins were aligned using MSAProbs, a new-generation alignment algorithm. This alignment was used to calculate of maximum-likelihood, neighbor-joining, and minimal-evolution phylogenetic trees. The common branch of animal and *Reticulomyxa* caspases was supported by 92–95% bootstrap values in different trees when viral and archaeal proteins were included in the analysis (Figure 2). These trees also indicated that the viral peptidases are closely related to the branch of animal caspases (with bootstrap values of 90–97%), suggesting that viruses hijacked caspase.

The third piece of evidence is provided by BLAST searches which revealed a close homology between caspases and the caspase-like proteins of *Reticulomyxa* (see Additional file 2). The close relationship between the animal and *Reticulomyxa* caspases suggests that there was a horizontal transfer of caspase genes. We tested this hypothesis using the chronology of phylogenetic events predicted by the RelTime method. Contrary to expectation, the splitting of animals and *Reticulomyxa* was the first event in the phylogenetic tree, it is therefore unlikely that an ancient horizontal transfer of caspase genes took places.

In conclusion, the results presented above suggest that the protomitochondrion encoded both caspases and metacaspases.

### Ancient Apoptotic Proteases – OMI/HTRA

HTRA proteases are involved in regulated proteolysis and quality control in both eubacterial and eukaryotic organisms (Clausen *et al.*, 2011). Some have an apoptotic function in Opisthokonta where they inhibit caspase apoptosis inhibitors containing BIR domains, the so-called inhibitor of apoptosis proteins, or IAPs (Suzuki *et al.*, 2001; Walter *et al.*, 2006). This inhibition of inhibitors activates apoptosis.

**Figure 3:**
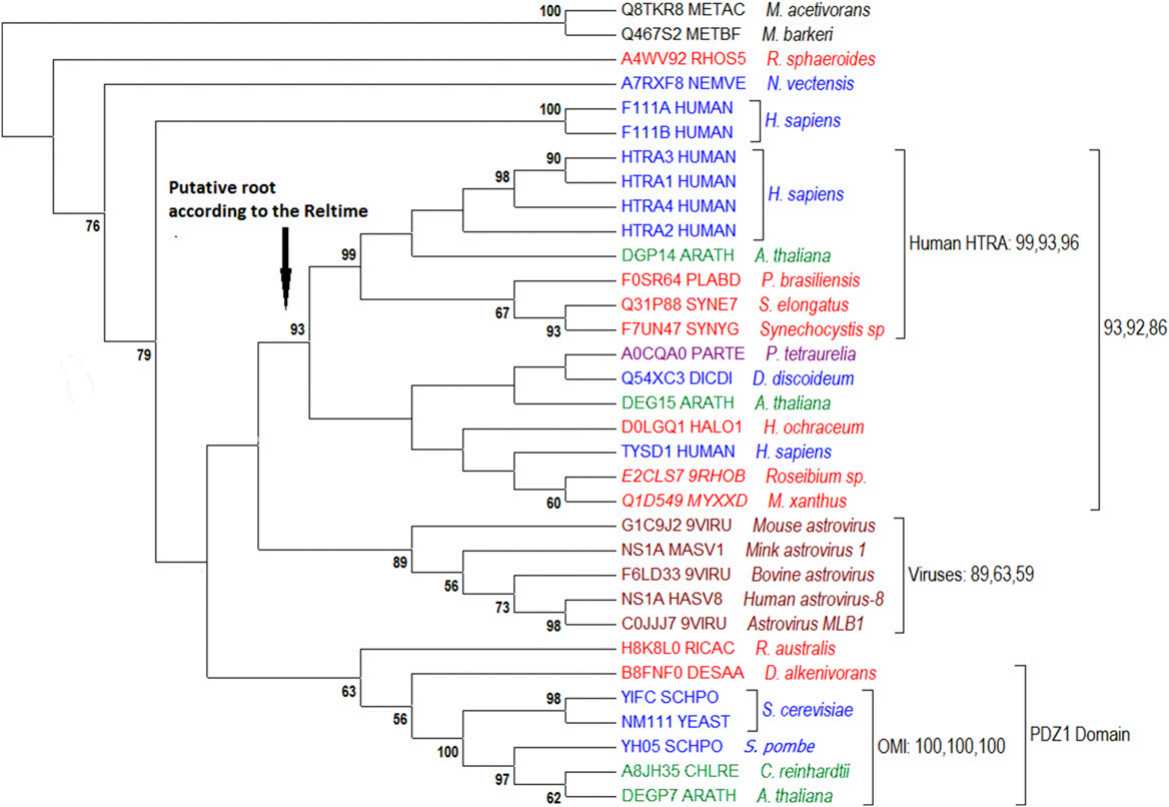
Phylogenetic tree of OMI/HTRA proteases. Three comma-separated bootstrap values (100 replicates) for the most significant branches were calculated with maximum likelihood estimation (MLE), neighbor-joining (NJ), and minimal evolution (ME). Red indicates bacterial proteins, black- Archaea, dark blue-Opisthokonta/Amoebozoa, green-Archaeplastida, purple-SAR, pink-Excavata, and brown-viral proteins.

Our sequence analysis revealed that these seemingly typical orthologous proteins diverged before the appearance of eukaryotes. This unexpected conclusion is based on different pieces of evidence. The first derives from an analysis of the phylogenetic tree of all 11,248 proteases belonging to the trypsin_2 family (those containing the Trypsin 2 ID Pfam domain PF13665; see Additional file 1). This domain is present in proteins from all branches of life: viruses, eubacteria, and archaea. The maximum likelihood tree (FastTree) revealed that human and yeast OMI/HTRA proteins are more closely related to eubacterial proteins than to each other or to any archaeal homologs. We further tested this hypothesis using different alignments of representative sequences, including archaeal and viral proteins, to which various tree-construction algorithms were applied (Figure 3). We found two independent ancient branches. The first unites yeast OMI/HTRA with putative chloroplast proteases of *Arabidopsis* and *Chlamydomonas*, with a bootstrap value of 100%. The second unites human OMI/HTRA proteins with putative bacterial serine proteases and a putative protease of *Arabidopsis*, with bootstrap values of 93–99%. Indeed, the human branch of OMI/HTRA is part of a larger branch with bootstrap values of 86–93%. This larger branch unites the human OMI/HTRA branch with other bacterial proteases and proteases from various major eukaryotic clades, namely SAR (*Paramecium*), Archaeplastida (*Arabidopsis*), Amoebozoa (*Dictyostelium*) and Opisthokonta (human TYSND1 protease). According to RelTime, this branch diverged at the beginning of the evolutionary history of OMI/HTRA proteases. Still, there are bacterial proteins that are more closely related to yeast OMI protease than to human HTRA protease. This hypothesis was additionally confirmed using BLAST searches (see Additional file 2). The fungal HTRA/OMI proteases contain additionally Pfam domain PDZ1, which is absent in the mammalian branch.

In conclusion, the results presented above suggest that fungal and animal HTRA/OMI proteases diverged before mitochondrial domestication.

### Components of Electron Transport Chain

The apoptotic function of cytochrome *c*, which is component of the respiratory chain, has been well demonstrated in Opisthokonta: animals (Zou *et al.*, 1999) and fungi (Silva *et al.*, 2005). When released from mitochondria, cytochrome c induces apoptosis. It is an evolutionarily conserved protein found in all branches of life.

Apoptosis-inducing factors (AIFs) are mitochondrial flavoproteins involved in oxidative respiration. Their apoptotic function has been shown in organisms belonging to different ancient eukaryotic phylogenetic branches including Opisthokonta (*e.g.*, human factors AIM1 (Susin *et al.*, 1999), AIM2 (Ohiro *et al.*, 2002), and AIM3 (Xie *et al.*, 2005), yeast factors NDI1 (Li *et al.*, 2006) and AIF1 (Wissing *et al.*, 2004) as well as SAR (AIF of Tetrahymena (Akematsu and Endoh 2010). All these proteins carry an oxidoreductase domain (Pyr_redox_2 domain, Pfam ID PF07992; see Additional file 1).

**Figure 4:**
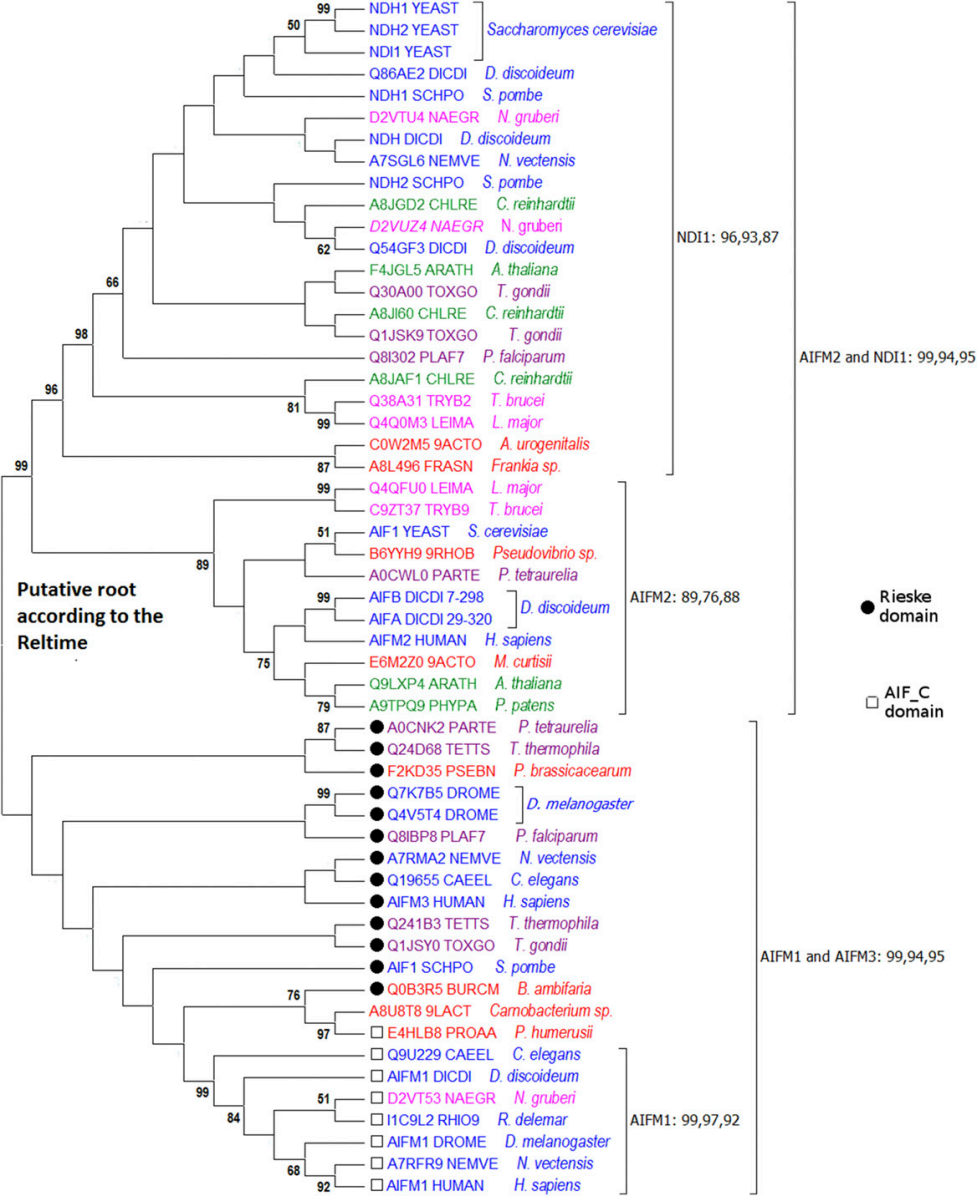
Phylogenetic tree of AIFs. Three comma-separated bootstrap values (100 replicates) for the most significant branches were calculated with maximum likelihood estimation (MLE), neighbor-joining (NJ), and minimal evolution (ME). Red indicates bacterial proteins, black-Archaea, dark blue-Opisthokonta/Amoebozoa, green-Archaeplastida, purple-SAR, pink-Excavata, and brown-viral proteins.

A detailed sequence analysis revealed that AIFs have a eubacterial origin and that many types of AIFs diverged before the origin of eukaryotes. Several pieces of evidence support this conclusion. Because the phylogenetic tree based on all 48,731 sequences in the Pfam database contained no monophyletic AIF branch, we calculated a tree using only 12,420 sequences belonging to branches containing different AIFs. The different AIFs were often more closely related to their bacterial homologs than to other eukaryotic AIFs. We identified five putative ancient branches of AIFs represented by human AIFs AIM1, AIM2, and AIM3 and two oxidoreductase domains of yeast NDI1 protein. We further tested this hypothesis using phylogenetic analyses of representative sequences from each of the three branches of human AIFs and the NDI branch. In each case and sequences belonging to each branch were included. The phylogenetic tree calculated for this subset is shown in Figure 4. There are two old branches, supported by strong bootstrap values (94–99%), each containing both eukaryotic and bacterial proteins branches. The chronology of evolutionary events predicted by RelTime places the root between these branches. The AIFM1/AIFM3 branch contains a clear AIFM1 sub-branch containing protein sequences from various ancient eukaryotic taxonomic clades: Excavata (*Naegleria*), Amoebozoa (*Dictyostelium*), and Opisthokonta (*Metazoa*) and supported by strong bootstrap values (97–99%). The NDI1 branch is supported by strong bootstrap values (93–99%) and contains both eubacterial proteins and sequences from the five major ancient eukaryotic groups (Opisthokonta, SAR, Archaeplastida, Excavata, and Amoebozoa). The AIFM2 branch, with lower bootstrap values of 76–89%, also contains both eubacterial proteins and proteins from the five ancient eukaryotic taxonomic groups.

In conclusion, the phylogenetic tree based on the alignment of a small number of representative sequences indicates that the divergence of the AIFM1, AIFM2, AIFM3, and NDI sequences occurred before the divergence of eukaryotes. This conclusion is supported by domain architecture of these proteins. Both eukaryotic and eubacterial AIFM1 proteins contain additional domain AIF_C, and AIFM3 contains the additional Rieskie domain. We also confirmed this conclusion using BLAST searches (see Additional file 2).

### Inhibitors of Apoptotic Proteases

We posit that protoeukaryotes developed inhibitors of bacterial toxins, which later evolved into apoptotic factors. We therefore asked which of the exclusively eukaryotic proteins could interfere with the apoptosis induced by the protomitochondrion. As mentioned above, proteins from the OMI/HTRA family induce apoptosis through degradation of the apoptosis inhibitors survivin/BIR1p in both animals and fungi (Verhagen *et al.*, 2002; Walter *et al.*, 2006). Given that our results suggested that fungal and animal OMI/HTRA proteases diverged before the origin of eukaryotes, a survivin-like anti-apoptotic protein was probably present in the ancestral state. We used sequence analysis to test this possibility.

Survivin proteins contain the BIR domain (Pfam ID PF00653) and belong to the IAP family (Verhagen *et al.*, 2001). We studied the evolutionary history of the BIR domain. Homology searches indicated that this domain is present almost exclusively in proteins of the Opisthokonta and viruses, but there are homologous proteins in other eukaryotic groups as well, namely SAR, Excavata and Archaeplastida (*N. gruberi* protein D2UXF5, *P. tetraurelia* A0CYG7, and *Guillardia theta* L1JDG1, respectively). We obtained more information about the evolutionary history of the BIR domain using an analysis of the clans (remotely related protein families). The BIR domain belongs to the BIR-like clan (Pfam ID CL0417), comprising remotely evolutionarily related protein domains RSM1 and zf-CH3. Proteins belonging to this clan are exclusively viral and eukaryotic and all the main eukaryotic taxonomic groups are represented.

The second group of putative ancient inhibitors of apoptosis is proteins similar to the extant AAC11 protein. AAC11 is an inhibitor of apoptosis overexpressed in several cancer lines and its down-regulation increases the sensitivity of cancer cells to various anticancer drugs. It has been shown that AAC11 interacts with the acinus (apoptotic chromatin condensation inducer) protein and prevents its caspase3-mediated cleavage (Rigou *et al.*, 2009) and, as a result, apoptosis. AAC11 contains the API5 domain (Pfam ID PF05918). Proteins containing this domain are absent in eubacteria, archaea and viruses, but they are encoded by genomes from several ancient eukaryotic taxonomic groups.

We conclude that the API5 inhibitor of caspase likely appeared before the divergence of the main eukaryotic taxonomic groups.

### Experimental Evolution

The results presented above suggest the following plausible scenario for the evolution of apoptosis. The eubacterial ancestors of mitochondria produced toxins that killed protoeukaryotes, therefore there was an antagonistic interaction between the protomitochondria and protoeukaryotes of a predator-prey or a host-parasite nature. It is not apparent, why the apoptosis machinery was not lost after the mitochondrial domestication and establishing of symbiosis. We postulate that because mitochondrial domestication was an adaptation to aerobic conditions also the apoptosis machinery was and still is involved in this adaptation. Negative pleiotropy results show that the apoptosis factors are toxic to eukaryotic cells and induce apoptotic cell death, but they are also involved in the adaptation to aerobic conditions. This hypothesis explains why apoptosis was not lost during mitochondrial domestication and why it is maintained in unicellular organisms, where no function in the regulation of developmental processes can be postulated. If this hypothesis is correct then deletion of apoptosis factors should be deleterious in aerobic conditions but beneficial in anaerobic conditions.

We tested this hypothesis experimentally using a yeast model (*S. cerevisiae*). As we showed at the beginning of this paper, the core apoptosis machinery in yeast comprises only five proteins belonging to ancient groups. It is expected that the apoptosis machinery of this ancient organism is still similar to the ancestral machinery which appeared during the mitochondrial domestication. We investigated proteins belonging to this core: NDI1, a mitochondrial apoptotic induction factor (AIF), NUC1, a mitochondrial apoptotic DNase ENDOG, MCA1, a cytoplasmic metacaspase and NMA111, a cytoplasmic apoptotic protease.

**Figure 5:**
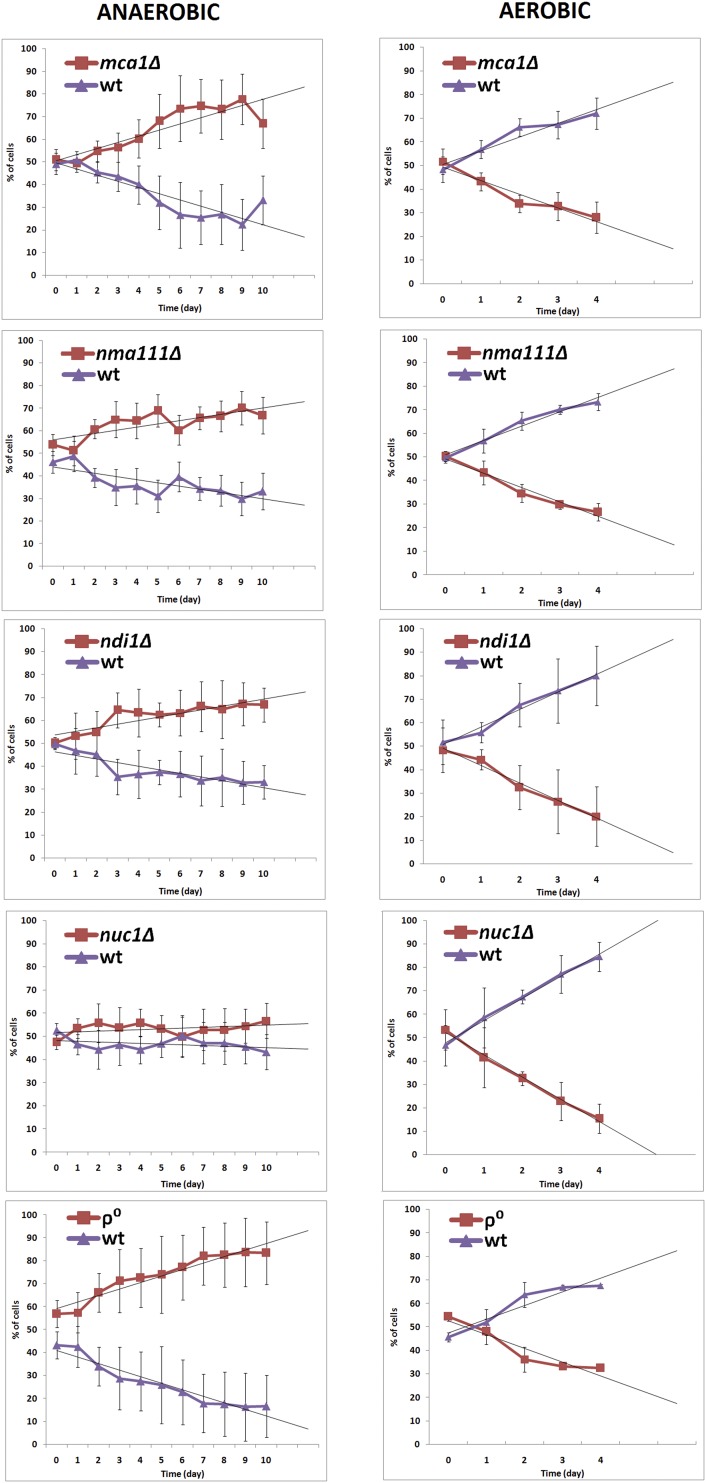
Competition of wild-type and mutant *S. cerevisiae* strains under anaerobic and aerobic conditions. Competition assays were performed between *ndi1Δ*, *nuc1Δ*, *mca1Δ*, *nma111Δ*, ρ^0^ and wild-type *S. cerevisiae* MR6 strains. The ratio of cells of the mutant and wild-type strains was monitored by plating ca. 100 cells onto YPD solid medium and replicating grown colonies onto YPD medium supplemented with geneticin or onto YPG to distinguish the mutant cells from the wild type. Data for each graph represent the mean ± SD of three independent assays.

To characterize the effects of deletion of those genes, we performed a simple competition experiment comparing the proliferation rates of isogenic wild-type and respective deletion-mutant yeast and ρ^0^ strains (incapable of mitochondrial respiration) co-cultivated in an anaerobic or aerobic atmosphere. Equal numbers of cells of both strains (from early exponential growth phase) were mixed and cultured in an O_2_-free or O_2_-containing liquid medium. Aliquots of each co-culture passage were plated on rich YPD medium and the numbers of colonies of the wild-type and mutant strains were determined. As shown in Figure 5, in anaerobic conditions the wild-type cells were gradually losing competition with *ndi1Δ*, *mca1Δ* and *nma111Δ* but not *nuc1Δ*. However, predicted trend-lines revealed that wild type cells are losing competition also in the case of *nuc1Δ*, although the observed advantage of the mutant was very weak.

In contrast, in aerobic conditions all the mutants studied were outperformed by wild type cells (Figure 5) confirming the earlier observation that these genes are required for efficient aerobic growth. The same behaviors were observed when a ρ^0^ derivative of MR6 strain served as a control, in both experimental conditions. Similar results were obtained when a pair of *S. cerevisiae* BY4741 (wt and *ndi1Δ)* strains were used in competition experiments (see Additional Figure S1).

The growth of all strains in aerobic and anaerobic conditions was also tested individually (see Supp. Figure S2). As expected from the competition experiments, in anaerobic conditions the wild-type strain grew slower and entered the stationary phase earlier than the mutants.

**Figure 6:**
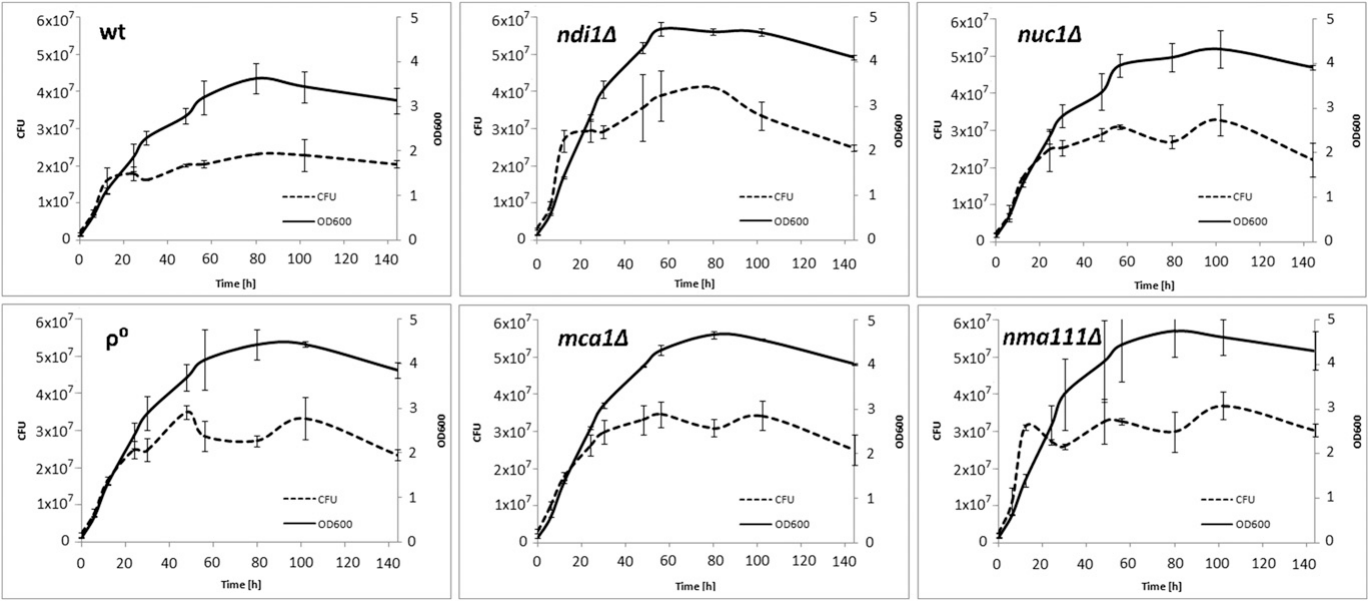
Effect of *nuc1*, *ndi1*, *mca1* or *nuc1* deletion on growth and viability of *S. cerevisiae* in prolonged cultures under anaerobic condition.Optical density (OD_600_) and density of live cells (CFU) were determined in parallel during culture growth. Mean and standard deviation from duplicate experiments are shown for each time point.

**Figure 7:**
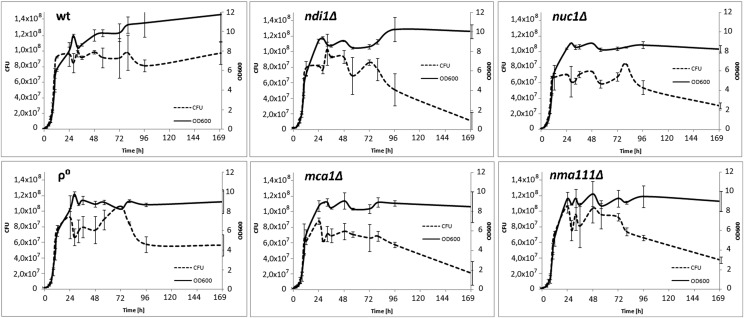
Effect of *nuc1*, *ndi1*, *mca1* or *nuc1* deletion on growth and viability of *S. cerevisiae* in prolonged cultures under aerobic conditions. Experiment was performed as described in legend to Figure 6.

The differences in fitness between the mutants and wild-type cells found in the competition experiments could result from differences in their proliferation rates and/or cell death rates. To differentiate between these possibilities we simultaneously monitored the optical density (OD) of cultures, which measures the number of all cells, and the number of colony forming units (CFU), which gives the number of live cells, for each strain. As shown in Figure 6 and 7, during the exponential growth phase the CFU and OD values were linearly proportional to each other in both aerobic and anaerobic conditions for all the strains, indicating identical death rates.

This experiment also explained, why the results obtained for the *nuc1Δ* mutant contradicted the earlier study by Bűttner and colleagues (Büttner *et al.*, 2007). Those authors found that deletion of *nuc1* stimulated necrosis in fermentative conditions. Apparently, under conditions used in our competition assays cell death was not stimulated.

In the stationary phase of growth (aging cultures) the curves differed substantially between anaerobic and aerobic conditions. In the former, the OD and CFU curves remained parallel, indicating no changes in the death rate during chronological aging in any of the strains (Figure 6). Thus, under anaerobic conditions deletion of the apoptotic genes does not affect the cell survival in the aging cultures. In contrast, under aerobic conditions the aging cultures of all the mutants studied, with the exception of the ρ^0^ strain, showed a steadily decreasing proportion of live cells (Figure 7). Thus, an absence of any of the apoptotic factors studied increases the death rate of chronologically aging cells in aerobic conditions.

To verify how the deletions studied affected the ability to perform mitochondrial respiration, we studied the growth of the respective yeast strains on a glycerol-containing medium. Glycerol is a non-fermentative carbon source, hence to use it as a sole source of energy yeast require mitochondrial respiration. In accordance with the literature data on the genes studied, deletion of any one of them precluded growth on the glycerol medium. Only the wild type strain grew on such medium (See Suppl. Figure S3.).

To conclude, these experiments confirmed our hypothesis that the apoptosis machinery is involved in adaptation of cells to aerobic conditions. In such conditions the activity of this machinery is beneficial both during exponential stage of culture growth and during chronological aging. Since the mitochondrial apoptotic factors (NDI1, NUC1) are required for proper mitochondrial functioning this observation was expected. Additionally, the results presented above suggest that apoptotic cytoplasmic proteases (OMI/HTRA and metasacpase) are also required for proper mitochondrial function.

## Discussion

**Figure 8:**
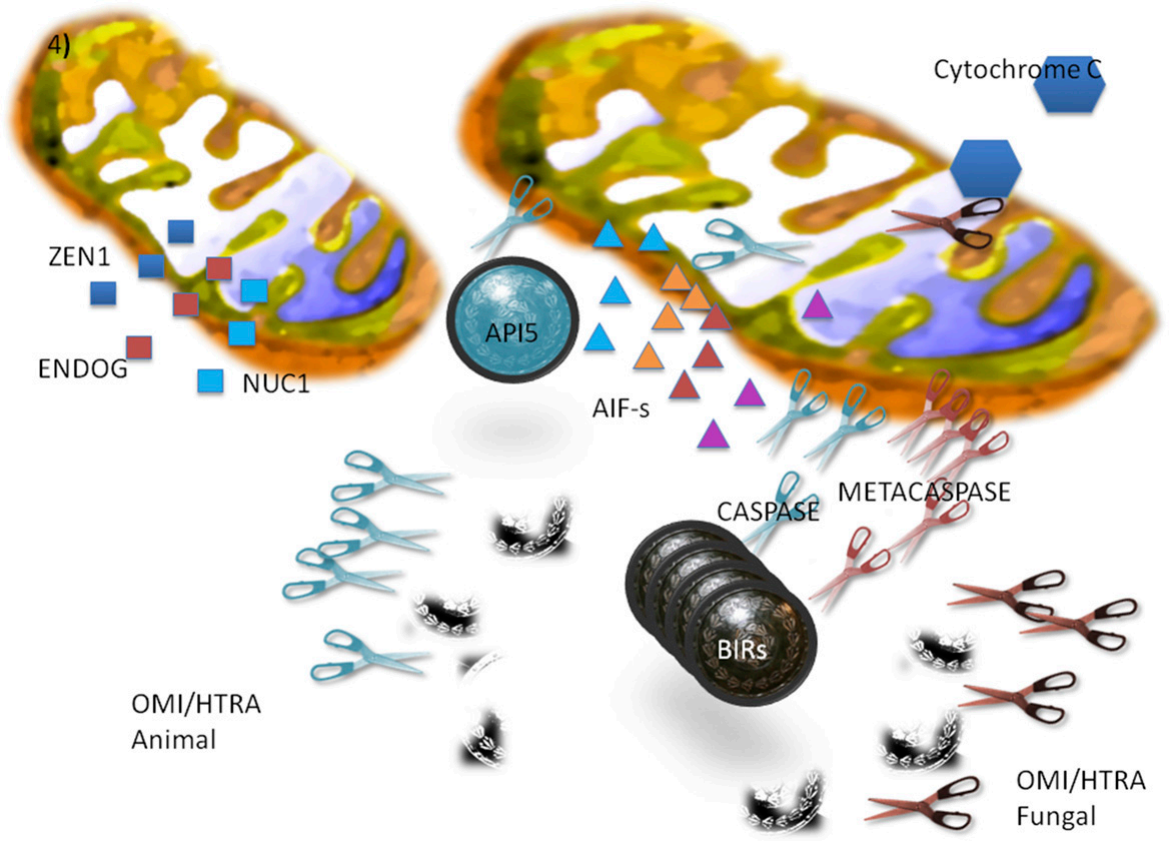
Ancestral state and evolutionary arms race leading to establishment of modern apoptosis machinery. According to this reconstruction, the parasitic protomitochondrion released the following toxins: DNases ZEN1, ENDOG and NUC1 (squares), diverse AIFs (marked as triangles), and different proteases (caspases and metacaspases, scissors). Inhibitors of the proteases, (depicted as shields: BIRs in black, API5-AAC11 in blue) represent the evolutionary response of the protoeukaryotic cell. The protomitochondrial proteases OMI/HTRA, which deactivate BIRs, are in turn an evolutionary response to the BIRs. As a result, two similar complex mechanisms evolved comprising nuclease, metacaspases or caspases, BIRs, and OMI/HTRA proteases. It is likely that the two systems diverged very early in evolution, before the divergence of the main eukaryotic groups. Image imported from Wikipedia (https://fr.wikipedia.org/wiki/Bouclier_(arme)#/media/File:4165_-_Milano_-_Antiquarium_-_Replica_armatura_gladiatore_-_Foto_Giovanni_Dall%27Orto_-_14-July-2007_-_1.jpg (Self-published work by G.dallorto).

Our analysis supports the endosymbiotic theory of the origin of apoptosis (Figure 8). Using yeast as a model we found that the apoptosis machinery of unicellular organisms is extremely simplified in comparison to animal ones. We focused on the reconstruction of the ancestral state of apoptosis mechanisms shared by eukaryotic unicellular and multicellular organisms.

The apoptosis system of the protoeukaryotes reconstructed using the parsimony assumption indicates that the putative ancestor had several apoptotic factors with redundant apoptotic functions, including apoptotic DNases (ZEN1, ENDOG, NUC1), caspase-type proteases (both metacaspase and metacaspase), various HTRA/OMI proteases (both fungal and mammalian types), and diverse AIFs. During subsequent evolution, redundant factors were lost in various lineages (for example, caspases and metacaspases, various DNases, or different HTRA/OMI proteases). Our analysis indicates that both ancient cytoplasmic and mitochondrial apoptotic factors have eubacterial origin. This supports the hypothesis that originally the apoptotic factors were toxins used by the ancestors of mitochondria against eukaryotic ancestors before mitochondrial domestication. Similar interactions between extant bacteria and eukaryotic cells are known, *e.g.*, bacterial proteases (Rust *et al.*, 2016) and DNases (Faïs *et al.*, 2016) are used as toxins inducing apoptosis. The observation that numerous apoptotic factors have eubacterial homologs and are found in different branches of the eukaryotic tree indicates that these factors are ancient and likely diverged before the origin of eukaryotes. Our analysis based on molecular clock excluded the possibility of horizontal transfer of the apoptotic factors between protomitochondria during the early stages of mitochondrial domestication.

In conclusion, apoptosis in its current complex form evolved by divergent evolution, as all the main apoptotic factors were already encoded in the protomitochondrion. However, the different apoptotic factors used by the protomitochondrion evolved by convergent evolution, evolution in the sense that several groups of unrelated factors become engaged in the same process (*e.g.*, the apoptotic DNases are not related to apoptotic proteases). Our study indicates that animal apoptosis and apoptosis/apoptosis-like cell death of plants and unicellular organisms are homologous processes. Indeed, we found that caspases are present in common ancestor of Eukaryotes. Such observation suggests that it is likely that the typical animal apoptosis based on centrality of activation of caspases evolved before animals.

A comparison with the phylogenetic studies performed by Koonin and colleagues 15 years ago reveals both advantages and limitations of the phylogenetic approach (Koonin and Aravind 2002; Aravind *et al.*, 2001). Our results support their hypothesis that apoptosis originated in eubacteria. However, they proposed that AIF originated in archaea because eubacterial homologs were not known at that time.

Some elements of the oxidative chain behaved as toxins toward protomitochondrion (such as various AIF oxidoreductases and cytochrome *c*). Interestingly, in different types of AIFs different domains have apoptotic activity, indicating that the mechanisms of apoptosis activation could also be divers (Kaczanowski 2016). The surprising richness of redundant apoptotic factors present in the protomitochondrion (for example, HTRA/OMI proteases) suggests that “red queen” co-evolution may have shaped the protomitochondrion to contain as many toxins as possible (van Valen 1973). This concept is nearly illustrated by extant parasites which often evade the host defense system by recombining numerous homologous pathogenic factors (*e.g.*, malarial *var* genes (Su *et al.*, 1995. It is difficult for the host to evolve resistance against many similar, but variable pathogenic factors. Our analysis suggests that a similar co-evolutionary arms race between ancestral eubacterial protomitochondrial and protoeukaryotic cells took place during the domestication of mitochondria. As to the putative antitoxins developed by the protoeukaryotic host of the protomitochondrion, we identified two plausible factors: the API5 and the IAP apoptosis inhibitors. Our phylogenetic analysis indicates that these factors could be very old and may have appeared when the symbiosis between protomitochondrial and protoeukaryotic organisms was being established.

In conclusion a co-evolutionary arms race likely contributed to the formation of the complex apoptotic regulatory pathways presented in Figure 8.

The experiments carried out in yeast confirmed a major predictions of the proposed model. They showed that perturbation of apoptosis could be beneficial in extant organisms in anaerobic conditions. We conclude that this result is a trace of the ancestral state and highlights the ancestral trade off. Mitochondrial domestication was beneficial in adapting to emerging aerobic conditions, and apoptotic-like cell death was the cost.

### Conclusion

In conclusion, our study suggests that the apoptosis machinery can be an ancient adaptation that evolved during mitochondrial domestication and is involved in adaptation to aerobic conditions in different eukaryotes.

Our results could have also medical implications. Since suppression of apoptosis leads to perturbation of mitochondrial metabolism in yeasts, one may expect that similar phenomenons exist in other eukaryotes. Actually, classical observations indicate that suppression of apoptosis in cancer cells tends to occur together with perturbations in cellular aerobic metabolism (the Wa; Warburg 1956). In contrast, pathological apoptosis in aging occurs in neuron cells during the course of Alzheimer’s (LaFerla *et al.*, 1995) and Parkinson’s (Mochizuki *et al.*, 1996)diseases, where mitochondrial respiration is extremely active (inverse Warburg hypothesis). Indeed, there is statistical important inverse epidemiological co-morbidity between these neurological diseases and cancer (Demetrius *et al.*, 2014; Demetrius and Simon, 2013, 2012).
